# The role of surgical tissue injury and intraoperative sympathetic activation in postoperative immunosuppression after breast-conserving surgery versus mastectomy: a prospective observational study

**DOI:** 10.1186/s13058-024-01801-0

**Published:** 2024-03-11

**Authors:** Lotte MC Jacobs, Leonie S Helder, Kim I Albers, Josephine Kranendonk, Christiaan Keijzer, Leo AB Joosten, Luc JA Strobbe, Michiel C Warlé

**Affiliations:** 1 Department of Surgery, Radboud University Medical Center, Geert Grooteplein zuid 10, Nijmegen, 6525 GA The Netherlands; 2 Department of Anaesthesiology, Radboudumc, Nijmegen, The Netherlands; 3 Department of Internal Medicine, Radboud Institute of Molecular Life Sciences, Radboudumc, Nijmegen, The Netherlands; 4 Department of Medical Genetics, Iuliu Hatieganu University of Medicine and Pharmacy, Cluj- Napoca, Romania; 5 Department of Surgery, Canisius Wilhelmina Hospital, Nijmegen, The Netherlands

**Keywords:** Breast-conserving surgery, Mastectomy, Immunosuppression, Surgical tissue injury, Sympathetic activation

## Abstract

**Background:**

Breast cancer is the second most common cause of death from cancer in women worldwide. Counterintuitively, large population-based retrospective trials report better survival after breast-conserving surgery (BCS) compared to mastectomy, corrected for tumour- and patient variables. More extensive surgical tissue injury and activation of the sympathetic nervous system by nociceptive stimuli are associated with immune suppression. We hypothesized that mastectomy causes a higher expression of plasma damage associated molecular patterns (DAMPs) and more intraoperative sympathetic activation which induce postoperative immune dysregulation. Immune suppression can lead to postoperative complications and affect tumour-free survival.

**Methods:**

In this prospective observational study, plasma DAMPs (HMGB1, HSP70, S100A8/A9 and S100A12), intraoperative sympathetic activation (Nociception Level (NOL) index from 0 to 100), and postoperative immune function (plasma cytokine concentrations and ex vivo cytokine production capacity) were compared in patients undergoing elective BCS (*n* = 20) versus mastectomy (*n* = 20).

**Results:**

Ex vivo cytokine production capacity of TNF, IL-6 and IL-1β was nearly absent in both groups one hour after surgery. Levels appeared recovered on postoperative day 3 (POD3), with significantly higher ex vivo production capacity of IL-1β after BCS (*p* = .041) compared to mastectomy. Plasma concentration of IL-6 was higher one hour after mastectomy (*p* = .045). Concentrations of plasma alarmins S100A8/A9 and S100A12 were significantly higher on POD3 after mastectomy (*p* = .003 and *p* = .041, respectively). Regression analysis showed a significantly lower percentage of NOL measurements ≤ 8 (absence of nociception) during mastectomy when corrected for norepinephrine equivalents (36% versus 45% respectively, *p* = .038). Percentage of NOL measurements ≤ 8 of all patients correlated with ex vivo cytokine production capacity of IL-1β and TNF on POD3 (*r =* .408; *p* = .011 and *r =* .500; *p* = .001, respectively).

**Conclusions:**

This pilot study revealed substantial early postoperative immune suppression after BCS and mastectomy that appears to recover in the following days. Differences between BCS and mastectomy in release of DAMPs and intraoperative sympathetic activation could affect postoperative immune homeostasis and thereby contribute to the better survival reported after BCS in previous large population-based retrospective trials. These results endorse further exploration of (1) S100 alarmins as potential therapeutic targets in breast cancer surgery and (2) suppression of intraoperative sympathetic activation to substantiate the observed association with postoperative immune dysregulation.

## Introduction

Breast cancer is the most common cancer among women and is the second most common cause of death from cancer among women in the world [1]. Several large population-based retrospective trials have investigated survival rates between breast-conserving surgery (BCS) and mastectomy. Most of these trials report superior survival in the breast-conserving surgery group [2–6]. However, it is critical to acknowledge the potential for selection bias inherent to these retrospective analyses, as they may not fully account for patient-specific factors such as mobility and frailty which can influence treatment decisions. This discovery, while reported consistently, is not geographically bound, nor age dependent, and has been adjusted for all tumor- and patient variables available in the cancer registration database [7]. Nonetheless, the retrospective nature of these trials necessitates a cautious interpretation of this association.

There is no straightforward explanation why limited surgery for early-stage breast cancer could lead to a better survival in comparison to mastectomy, and thus these findings should be considered hypothesis-generating rather than conclusive. A possible contributing factor to this observation is radiotherapy, since the majority of patients undergoing BCS receive radiotherapy, as opposed to fewer patients after mastectomy [8]. Furthermore, there is a larger degree of surgical trauma in mastectomy, which is associated with increased odds of developing a postoperative complication [9]. Complication rates are already low after breast cancer surgery. Still, a recent study also showed that mastectomy has higher medical and surgical postoperative complication rates than BCS [10]. A plausible hypothesis emerging in recent literature is that more extensive surgical trauma could lead to immune dysregulation. Cell damage leads to the release of damage-associated molecular patterns (DAMPs), substances either actively released by cells under threat, or components of the cell exposed when a cell is injured [11, 12]. DAMPs function as ligands for Toll-like receptors that, upon binding, induce inflammation followed by a compensatory state of immune suppression [11, 13]. In addition, activation of the sympathetic nervous system by nociceptive stimuli is known to induce immune suppression [14, 15]. More surgical trauma could lead to more pain and a higher degree of intraoperative sympathetic activation, which can easily be quantified with a non-invasive monitor [16]. Fragidiakis et al. describe a strong correlation between immune status and recovery from surgery [17]. In trauma patients, the suppressed immune state has been linked to infectious complications and mortality [18–20].

Several DAMPs have been linked to postoperative immune suppression and infectious complications (High mobility group box 1 (HMGB1) and Heat shock protein 70 (HSP70)) [21], and breast cancer (alarmins S100A8/A9 and S100A12) [22–24]. While functions and interactions of individual DAMPs have not been comprehensively characterized, in general HMGB1 and HSP70 reflect the degree of surgical injury or tissue damage, while increased S100A8/9 and S100A12 are associated with morbidity and mortality [25] and cancer progression [26–30]. The aim of this pilot study was to further explore the role of these DAMPs and intraoperative sympathetic activation in immune suppression after breast cancer surgery, and to investigate whether these factors are likely to contribute to the previously reported difference in survival between BCS and mastectomy. We hypothesized that more extensive surgical trauma and sympathetic activation during mastectomy are associated with more immune suppression, which in turn may lead to postoperative complications and affect survival.

## Materials and methods

### Study population

Women undergoing elective BCS or mastectomy at the Canisius Wilhelmina Hospital (CWZ) in Nijmegen in the Netherlands were included in this single-centre prospective pilot study in 2021–2022. Patients were excluded if they were under 18 years of age or unwilling to give informed consent. The study protocol was approved by the Medical Research Ethics Committee “CMO region Arnhem-Nijmegen” (NL65918.091.18). Randomization was not applicable as only patients who were already scheduled to undergo either BCS or mastectomy were included. Participation in the study did not alter or delay the already scheduled treatment in any way. Written informed consent was obtained from all study participants before the start of any study-related procedures. Determinations and data handling were performed in agreement with the guidelines of The National Institutes of Health and in accordance with the declaration of Helsinki and its later amendments.

### Sample and data collection

Baseline-, tumour-, and treatment characteristics and perioperative parameters were obtained from digital patient files in the programme Healthcare Information eXchange (HiX). Pain scores (Numerical Rating Scale (NRS), 0–10, 10 worst pain score)) at one hour after surgery were obtained by attending nurses at the recovery. Blood samples were taken before surgery, 1 hour after surgery, and 3 days after surgery. Lithium heparin (LH) anti-coagulated blood was drawn for ex vivo endotoxin stimulation of leukocytes immediately after sampling and ethylenediaminetetraacetic acid (EDTA) anti-coagulated blood was obtained to measure plasma DAMP levels and cytokine concentrations. After blood withdrawal, both LH and EDTA anti-coagulated blood samples were centrifuged at 1,600 RCF at 4 °C for 10 min. EDTA anti-coagulated plasma samples were centrifuged again at 16,000 RCF at 4 °C for 10 min to remove potential remaining cells and cell debris. Plasma was stored at -80 °C until further analysis.

### Plasma DAMP and cytokine concentrations

Plasma concentrations of HMGB1 were measured batchwise using the HMGB1 Express ELISA according to the manufacturers protocol (TECAN, Männedorf, Switzerland, catalogue number 30,164,033). HSP70, S100A8/A9, and S100A12 plasma concentrations were measured using Human HSP70/HSPA1A, Human S100A8/S100A9 Heterodimer, and Human EN-RAGE DuoSet ELISAs according to the manufacturer’s protocol (R&D systems, Minneapolis, MN, USA, catalogue numbers DY1663-05, DY8226-05, and DY1052-05 respectively). Concentrations of TNF, IL-6 and IL-10 were measured batchwise in plasma from EDTA anti-coagulated blood using a simultaneous Luminex assay (Milliplex; Millipore, Billerica, MA) according to the manufacturer’s instructions.

### Ex vivo cytokine production upon whole blood stimulation

Ex vivo cytokine production capacity upon lipopolysaccharide (LPS) stimulation was measured as previously described [19, 21, 31]. In short, 0.5 mL whole LH anti-coagulated blood was added to tubes with 2 mL Roswell Park Memorial Institute (RPMI) medium as negative control or 2 mL RPMI culture medium supplemented with 12.5 ng/mL *Escherichia coli* LPS (serotype O55:B5 Sigma Aldrich, St Louis, MO, USA), end concentration 10 ng/mL. These tubes were incubated at 37 °C with 5% CO_2_ for 24 h. Next, the samples were centrifuged at 3000 RPM and 24 °C for 5 min and supernatants were stored at -80 °C until further analysis. Concentrations of inflammatory cytokines IL-1β, TNF, IL-6 and anti-inflammatory cytokine IL-10 in the supernatants were measured using Human Bio-Techne R&D DuoSet ELISA according to the manufacturer’s protocol (R&D systems, Minneapolis, MN, USA). The plates were read at 450 nm using a ELx808 BioTek plate reader.

### Anaesthesia and nociception level

Anaesthesia was given according to the local standardized hospital protocol for breast surgery and consisted of total intravenous anaesthesia (TIVA) with remifentanil and propofol. Analgesia consisted of acetaminophen, diclofenac and intravenous morphine. During surgery, all patients were connected to a Nociception Level (NOL) monitor (Medasense Biometrics Ltd, Ramat Gan, Israel) by a finger probe that can detect and quantify mild to intense noxious stimulation. Using a validated algorithm, this monitor combines the physiological parameters heart rate, heart rate variability, plethysmograph wave amplitude, skin conductance level, number of skin conductance fluctuations, and their time into a single index: the NOL index [32]. Index values ranging from 0 (no nociception) to 100 (extreme nociception) were measured every 5 seconds and collected on the hard disc of the monitor until they were extracted for analysis. Generally, NOL values below 10 are considered absence of nociception [33, 34]. As median NOL values of 11–13 are reported during surgery (34), a NOL ≤ 8 was chosen as the cut-off value for this trial. The anaesthesiologist was blinded to the NOL index, measurements were not used to guide treatment in any way. Use of vasopressors (ephedrine, phenylephrine and norepinephrine were converted to norepinephrine equivalents [35]) was recorded to correct for increases in the NOL index secondary to the resulting increase in blood pressure and/or heart rate in ANCOVA.

### Statistical analysis

This pilot study will be used to determine estimated effects of BCS and mastectomy on measures of interest and plan subsequent studies. Therefore, no sample size calculation was performed. Data presented in tables and text are presented as mean with standard deviation (SD). Data presented in figures are expressed as mean with standard error of the mean (SEM). Independent samples T-tests and chi-squared tests were used to determine differences between the groups (BCS versus mastectomy) for each of the time points. Linear regression analysis was used to determine differences in nociception level index corrected for use of vasopressors. Furthermore, repeated measures ANOVAs with Bonferroni correction were performed to determine differences between the different timepoints within each group. Cytokine levels below the detection limit as determined by ELISA were considered equal to the lowest detectable concentration because these values were expected to be very low. Haemolytic blood samples were excluded from the analysis of DAMP levels because haemolysis has been described to influence the accuracy of the results and the reliability of laboratory testing [36, 37]. Correlations were determined using Pearson’s correlation.

All figures were made using GraphPad Prism version 5.03 and statistical analyses were performed using GraphPad Prism version 9.4.1 and SPSS version 27. P-values ≤ 0.05 were considered statistically significant.

## Results

### Patient characteristics

From February 2021 until June 2022, 20 women undergoing BCS and 20 women undergoing mastectomy at the Canisius Wilhelmina Hospital in the Netherlands were included in this study. Baseline characteristics age, BMI, and ASA score were similar between groups (Table 1). Incidence of invasive lobular carcinoma was more prevalent in the mastectomy group while invasive carcinoma of no special type (NST) was more prevalent in the breast-conserving surgery group. Neo-adjuvant chemotherapy occurred more frequently in the mastectomy group while frequency of administration of hormone therapy was similar between groups.

**Table 1:** Baseline characteristics

		Breast-conserving surgery (*n* = 20)	Mastectomy (*n* = 20)	P
**Patient characteristics**				
Age (years)		67.7 ± 9.0	63.1 ± 12.7	0.193
BMI (kg/m^2^)		27.8 ± 4.9	26.1 ± 4.5	0.257
ASA (I / II / III)		6 / 13 / 1	5 / 14 / 1	0.770
**Tumour characteristics**				
Indication for surgery				**0.019**
Invasive carcinoma NST Invasive lobular carcinoma Carcinoma in situ		19 0 1	12 6 2	
Unilateral / bilateral		19 / 1	19 / 1	1.000
Sentinel node excision (Y / N)		15 / 5	16 / 4	0.705
Oestrogen receptor (+/-/unknown)		18 / 2 / 0	16 / 3 / 1	0.589
Progesterone receptor (+/-/unknown)		15 / 5 / 0	14 / 5 / 1	0.925
Her2Neu receptor (+/-/unknown)		2 / 18 / 0	1 / 15 / 4	0.686
**Additional treatment**				
Neo-adjuvant chemotherapy (Y / N)		0 / 20	6 / 14	**0.008**
Letrozole (Y / N)		4 / 16	4 / 16	1.000

ASA = American Society of Anaesthesiologists classification, BMI = body mass index, NST = no special type

### Anaesthesia and pain

Total doses of anaesthesia and analgesia in units per hour administered during surgery did not differ between the groups (Table 2). However, duration of surgery was significantly longer for mastectomy compared to breast-conserving surgery (89 versus 58 min, *P* < .001). Pain and morphine consumption at the recovery room were significantly higher after mastectomy (Table 2).

**Table 2:** Anaesthesia and pain

	Breast-conserving surgery (*n* = 20)	Mastectomy (*n* = 20)	P
Duration of surgery (min)	58 ± 20	89 ± 27	**< 0.001**
Propofol (mg/kg/h)	9.7 ± 2.1	9.4 ± 1.9	0.692
Propofol (mg/kg IBW/h)	12.6 ± 2.4	11.6 ± 1.9	0.157
Remifentanil (ug/kg/h)	10.2 ± 3.7	10.8 ± 3.0	0.570
Remifentanil (ug/kg IBW/h)	13.2 ± 4.4	13.3 ± 3.5	0.866
Lidocaine (mg)	46	58	0.226
Ketamine (mg)	1	4	0.137
Ropivacaine (mg)	38	44	0.371
Morphine OR (mg)	3.5	4.3	0.237
Norepinephrine equivalents (ug/kg IBW/h)	1.2 ± 0.9	1.0 ± 1.1	0.608
iv. morphine equivalents OR (fentanyl + remifentanil + morphine in mg/kg IBW/h) [38]	1.4 ± 0.4	1.4 ± 0.3	0.990
Pain at the recovery room (NRS)	2.0 ± 1.1	3.5 ± 1.8	**0.004**
Morphine at the recovery room (IV in mg)	0.9 ± 2.0	3.4 ± 4.5	**0.037**

IBW = ideal body weight, IV = intravenous, NRS = numeric rating scale, OR = operating room

### Innate immune function

**Fig. 1:**
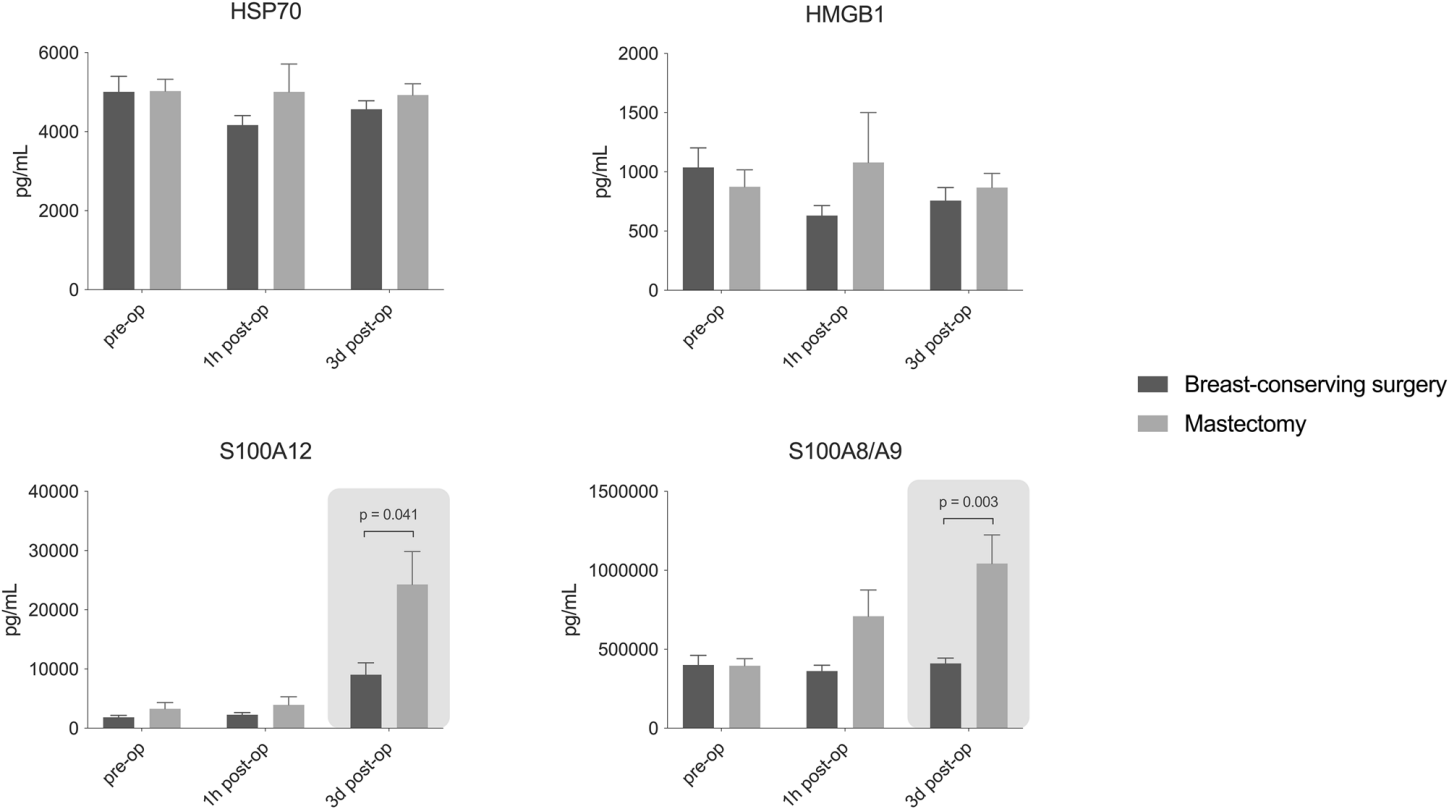
Concentrations of damage associated molecular patterns (DAMPs) in plasma before surgery, one hour (1 h) and three days (3d) after surgery. Data are presented as mean ± standard error. HSP70: heat shock protein 70, HMGB1: high mobility group box 1, S100A12: S100 calcium-binding protein A12, S100A8/A9: calprotectin

**Fig. 2:**
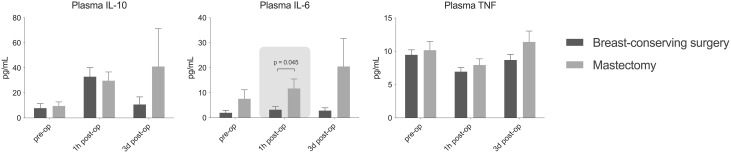
Plasma cytokine concentrations before surgery, one hour (1 h) and three days (3d) after surgery. Data are presented as mean ± standard error. IL: interleukin; TNF: tumour necrosis factor

**Fig. 3:**
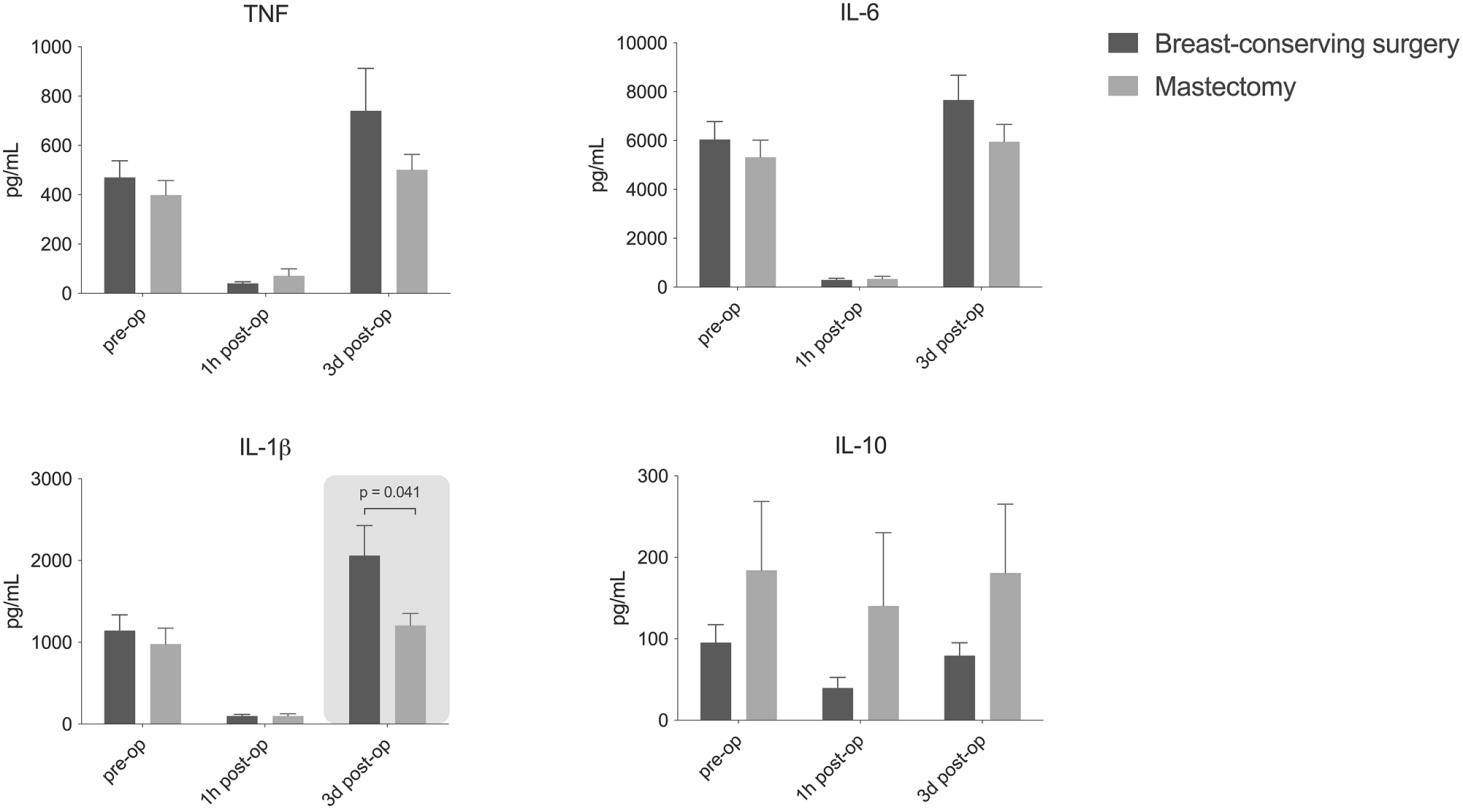
Ex vivo **cytokine production capacity of leukocytes upon whole blood stimulation with** ***E. coli*** **lipopolysaccharide (LPS) before, one hour (1 h) and three days (3d) after surgery. Data are presented as mean ± standard error. IL: interleukin; TNF: tumour necrosis factor**

Concentrations of DAMPs in plasma did not differ between the groups at baseline and one hour after surgery. However, on postoperative day 3 (POD3), concentrations of S100A8/A9 and S100A12 were significantly higher in patients after mastectomy (Fig. 1). Plasma concentrations of IL-6 were significantly higher one hour after mastectomy compared to BCS (Fig. 2). No other differences in plasma cytokine concentrations were detected between groups. In all patients collectively, strong immunosuppression was observed at one hour after surgery indicated by a significant decrease to nearly absent ex vivo production capacity of TNF, IL-6 and IL-1 β. Ex vivo cytokine production capacity appeared restored three days after surgery, ex vivo production of IL-1β upon endotoxin stimulation was significantly higher in the mastectomy group on POD3 (Fig. 3). No baseline differences were found for the assessed parameters between patients with and without neo-adjuvant chemotherapy, exclusion of patients with neo-adjuvant chemotherapy did not alter the results.

### NOL index

The mean observed NOL index for all 40 patients combined was 13 ± 5. The percentage of NOL measurements ≤ 8 correlated with ex vivo cytokine production capacity of IL-1β and TNF on POD3 (*r =* .408, *p* = .011 and *r =* .500, *p* = .001, respectively). Linear regression analysis shows patients undergoing mastectomy had a significantly lower percentage of NOL measurements ≤ 8 compared to patients undergoing BCS (36% versus 45%, respectively, *p* = .038) when corrected for norepinephrine equivalents by ANCOVA. Patients who developed a wound infection within 30 days after surgery (*n* = 3, one in the BCS group and two in the mastectomy group) had a significantly lower percentage of NOL measurements ≤ 8 (19% versus 42%, *p* = .023). The mean NOL and percentage of NOL measurements ≤ 8 did not correlate with pain scores at the postanaesthetic care unit (PACU).

## Discussion

In this pilot study, the role of DAMPs and intraoperative sympathetic activation in postoperative immune suppression after breast cancer surgery were explored to investigate whether these factors are likely to contribute to greater survival after BCS compared to mastectomy, as reported in previous large population based retrospective trials.

Plasma alarmins S100A12 and S100A8/A9 were significantly increased on postoperative day 3 after mastectomy as compared to BCS. During inflammation, S100A12 and S100A8/A9 are known to modulate the immune inflammatory response by stimulation of leukocyte recruitment and induction of cytokine secretion [39, 40]. The increased levels after mastectomy could be related to wound healing as there is a greater wound surface after mastectomy. Considering the association of high S100A12 and S100A8/A9 with worse prognosis in breast cancer and other types of cancer [25–29], this could be a contributing factor to the observed difference in survival in previous observational studies. Regulation of S100 alarmins as therapeutic targets in inflammatory disease is an upcoming area of research [41]. For both groups, HMGB1 and HSP70 were not significantly increased one hour and 3 days after surgery compared to before surgery. This was an unexpected finding, as several previous studies report an increase in these DAMPs after different types of surgery [21, 42–44]. Either the expression of these DAMPs is simply not increased after breast cancer surgery, or possibly the window of increase was outside the measured timepoints. There were also no differences between BCS and mastectomy for HMGB1 and HSP70.

Plasma IL-6 was significantly higher 1 hour after surgery for the mastectomy group. IL-6 is a major regulator of myeloid-derived suppressor cells (MDSCs) that suppress the anti-tumour functions of T and NK-cells. High plasma IL-6 is therefore associated with a worse prognosis as it leads to increased tumour cell proliferation and metastasis [45–47]. Additionally, more extensive surgical trauma and sympathetic activation during mastectomy may cause greater adrenergic and prostaglandin responses, which also affect immune function. Acute stress can promote the release of pro-inflammatory cytokines, such as IL-6 and IL-1β, while chronic stress can also contribute to immunosuppression [48]. This is in line with the current study as higher NOL values and higher plasma concentrations of IL-6 1 hour after mastectomy were found compared to breast-conserving surgery. Plasma TNF and IL-10 were not significantly different between BCS and mastectomy 1 hour and 3 days after surgery.

It is well recognized that the early postoperative immune response is predominantly immunosuppressive [49]. Leijte et al. [21] and Albers et al. [44] have previously described the association between DAMPs and ex vivo cytokine production capacity. Nonetheless, the fact that the ex vivo cytokine production capacity (regarded as the capacity to elicit an inflammatory response when encountering a pathogen [50]) is nearly absent one hour after surgery has not been described before. The observed levels were comparable to levels in patients displaying a state of immunoparalysis after sepsis [50, 51]. Whether this is the direct result of anaesthetics or intraoperative circulating DAMPs is still unknown. Anaesthetics commonly used in surgery have a direct effect on the functions of immunocompetent cells [52]. Propofol impairs several monocyte and neutrophil functions and remifentanil also presents strong immunomodulatory effects [53]. In general, the effects of anaesthetics appeared to be moderate to even negligible to the effects of surgical trauma in healthy patients anesthetized for short procedures [53, 54]. However, the newly identified tremendous depression of cytokine production capacity directly after surgery in patients with cancer and often other comorbidities perhaps calls for a re-evaluation of the role of anaesthetics in postoperative complications. Curiously, while ex vivo production capacity of IL-1β is approximately back to baseline on POD3 for mastectomy, it is significantly higher on POD3 after BCS. IL-1β, being a potent pro-inflammatory cytokine crucial to the host-defence against invading pathogens, is usually not detectable in plasma of healthy individuals [55, 56]. Higher production capacity upon endotoxin stimulation therefore indicates improved protection against postoperative infectious complications. IL-1β appears to have opposing functions in breast cancer, as high levels in the tumour micro-environment inhibit tumour growth while on the other hand it seems to enhance metastasis in bone [57]. Altogether, this study investigates only a small subset of DAMPs and a fraction of the innate immune response. Considering the small sample size and the pilot character of the study there is no foundation for hard conclusions. Also, our ex vivo findings should be interpreted with caution as this approach does not take in to account all factors present in the in vivo situation, such as stress hormones [58]. Nonetheless, the significant immune-related differences identified between BCS and mastectomy do suggest the type of surgery is substantive for the postoperative course and prognosis.

Intra-operative activation of the sympathetic nervous system (e.g. stress or nociception during general anaesthesia) could very well also contribute to the observed differences between BCS and mastectomy. More extensive surgical tissue injury understandably leads to more nociceptive activation. Morisson et al. previously described that the percentage of NOL measurements < 10 during surgery was predictive for pain at the PACU [34]. We did not find the same association for pain scores in the recovery room. However, the percentage of NOL measurements ≤ 8 during surgery did correlate with the ex vivo cytokine production capacity of IL-1β and TNF on POD3. Moreover, patients undergoing mastectomy had a significantly higher percentage of NOL measurements above this nociception threshold. While numbers are very small, patients who suffered a postoperative wound infection (*n* = 3) had a significantly lower percentage of NOL measurements ≤ 8. The current manufacturer guidelines direct that a NOL index of 0–25 represents an appropriately suppressed physiological response to noxious stimuli and adequate analgesia [59]. While the manufacturer’s instructions suggest a prolonged NOL < 10 may indicate excessive analgesia, these results and results of Morrison et al. support that striving for a lower threshold for absence of nociception could improve clinical outcomes. The connection between early postoperative pain and infectious complications is well established in breast cancer- and other types of surgery [60–62]. The optimal depth of anaesthesia as quantified by the bispectral index (BIS) had been long debated before the Balanced Anaesthesia. A previous study revealed no differences in one year mortality or severe adverse events between light and deep anaesthesia (BIS 50 versus 35, respectively) [63]. Relevant differences may however be present for the analgesia pillar of the triad of anaesthesia. These data support that a lower intraoperative NOL index is associated with less postoperative immune suppression and could lead to a lower risk of postoperative infections. A randomized trial investigating whether enhanced suppression of nociception with multimodal analgesia can reduce postoperative immune suppression is warranted.

There are certain limitations of this study that readers need to consider when interpreting the results. First, this study does not discriminate between the effect of surgical tissue injury and the duration of surgery. An extensive meta-analysis revealed that the likelihood of complications increased significantly with prolonged operative duration [64]. However, as no differences in the use of anaesthetics were found between the groups, we consider the larger extent of surgical tissue injury the most important consequence influence immune function due to longer duration of surgery. Second, this study did not take into account the effect of radiation, which can also influence postoperative survival.

In conclusion, our study has identified potential differences between BCS and mastectomy in the release of DAMPs and intraoperative sympathetic activation. These differences may influence postoperative immune homeostasis. While these findings could provide a partial explanation for the improved survival outcomes associated with BCS as observed in previous large population-based retrospective trials, it is imperative to recognize the limitations inherent in such retrospective analyses, including possible selection bias and the confounding role of adjuvant therapies. Therefore, our results should be interpreted as preliminary evidence that contributes to a growing body of research. These results endorse further exploration of (1) S100 alarmins as potential therapeutic targets in breast cancer surgery and (2) suppression of intraoperative sympathetic activation to substantiate the observed association with postoperative immune dysregulation.

## Data Availability

The datasets used and/or analysed during the current study are available from the corresponding author on reasonable request.
